# Trastuzumab Associated With Recurrent Severe Thrombocytopenia and Successful Use of Pertuzumab Monotherapy

**DOI:** 10.7759/cureus.21748

**Published:** 2022-01-30

**Authors:** Mallorie L Huff, Joshua A Kalter, Ramona E Chase, Ranju Gupta

**Affiliations:** 1 Division of Hematology and Medical Oncology, Morsani College of Medicine, University of South Florida, Tampa, USA; 2 Division of Hematology and Medical Oncology, Lehigh Valley Health Network, Allentown, USA

**Keywords:** her-2-neu, her-2 positive breast cancer, pertuzumab, perjeta, thrombocytopenia, herceptin, trastuzumab

## Abstract

Trastuzumab is a mainstay chemotherapeutic agent used in the treatment of human epidermal growth factor receptor 2 (HER2)/neu-positive breast cancer that, though generally well-tolerated, is classically associated with side effects like cardiotoxicity. Cytopenias can be seen but are generally secondary to other chemotherapeutic agents used in conjunction with trastuzumab. Herein, we present a case of recurrent severe thrombocytopenia following trastuzumab use that resolved following discontinuation. Our patient then finished a year of maintenance therapy with pertuzumab alone and is still in remission four years later. This is the eleventh report of this severe adverse effect described in the literature. This report contributes to the body of work describing this severe side effect by illustrating a clear temporal relationship between trastuzumab and severe thrombocytopenia, while also providing an alternate treatment option with chemotherapy and pertuzumab monotherapy. Given that pertuzumab is typically only used in addition to trastuzumab, evidence of its successful independent use is of clinical value to patients who may not be able to tolerate trastuzumab.

## Introduction

In the United States, breast cancer is the most common and second most lethal cancer in women. While numerous evidence-based guidelines exist for breast cancer, selecting treatment regimens is nuanced and complex, especially as expected toxicity or individual patient intolerances may necessitate modifications to chemotherapeutic dosing, agent, and schedule [1]. Trastuzumab and pertuzumab are mainstays of therapy in human epidermal growth factor receptor 2 (HER2)-positive breast cancer. Mechanistically, trastuzumab and pertuzumab are human epidermal growth factor receptor 2 (HER2) monoclonal antibodies that cosuppress HER2 activity through distinct binding sites and induce antibody-dependent cell-mediated cytotoxicity [2]. Dual anti-HER2 therapy has a profound mortality benefit and decreases risk of recurrence in both early [2] and metastatic disease [3]. Trastuzumab monotherapy in the adjuvant setting is similarly beneficial. However, the role of pertuzumab monotherapy in breast cancer is less clear. The standard of care for HER2-positive breast cancer is chemotherapy with a year of adjuvant anti-HER2 therapy with trastuzumab or an FDA-approved biosimilar agent with or without pertuzumab [1]. Trastuzumab is generally well-tolerated but may cause significant cardiotoxicity, especially when administered concurrently with anthracyclines [1,3]. Given the omnipresence of trastuzumab in neoadjuvant and adjuvant HER2-positive breast cancer therapy, it is essential to identify viable alternatives for patients who may not tolerate trastuzumab. In this report, we describe a significant trastuzumab intolerance with severe recurrent thrombocytopenia and describe the successful use of pertuzumab monotherapy in the adjuvant period.

## Case presentation

**Figure 1:**
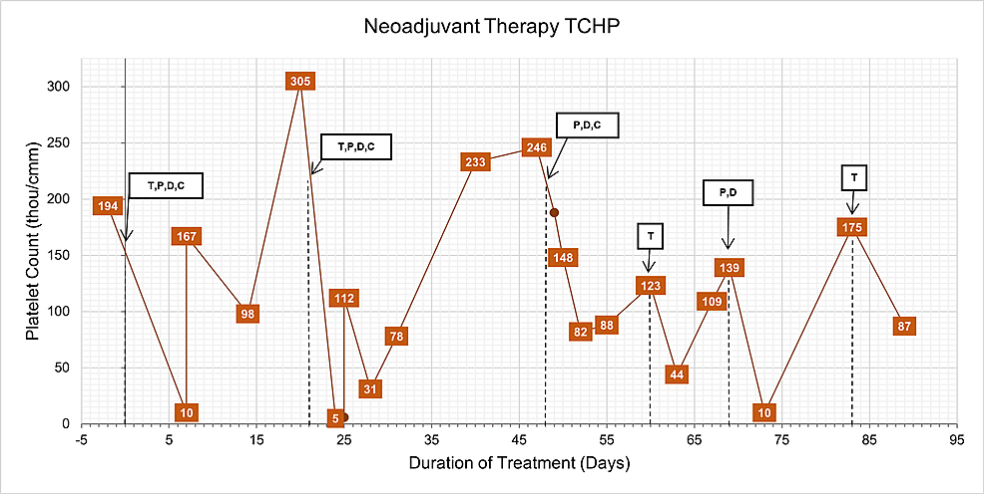
Graph of platelet count over treatment duration with overlayed neoadjuvant therapy timeline with trastuzumab, pertuzumab, docetaxel, and carboplatin administration. Dashed lines indicate when chemotherapeutic agents were administered and correspond with labels above. TCHP: Taxotere® + Carboplatin + Herceptin® + Perjeta®, T: trastuzumab, P: pertuzumab, D: docetaxel, C: carboplatin.

A 36-year-old, gravida 3, para 2 (G3P2), premenopausal woman with no significant medical history and menarche at age 14 presented with a one-month history of right upper outer quadrant breast fullness with gradual nipple retraction. Needle core biopsy showed a grade 3 invasive ductal carcinoma (IDC) that was weakly estrogen receptor (ER) and progesterone receptor (PR) positive (ER/PR 2%) and strongly HER-2/neu receptor positive (IHC 3+). Elective genetic testing for breast cancer type 1 susceptibility gene (BRCA1) and breast cancer type 2 susceptibility gene (BRCA2) was negative. Clinically, she was found to have T2N2M0 IDC measuring 4 cm on MRI with multiple (>4) abnormal right axillary lymph nodes identified on MRI and positron emission tomography (PET) scan. Neoadjuvant chemotherapy was initiated with docetaxel (Taxotere), carboplatin (Paraplatin), trastuzumab (Herceptin), and pertuzumab (Perjeta) (Taxotere® + Carboplatin + Herceptin® + Perjeta® (TCHP) regimen) (Table 1). Prior to beginning her first cycle of TCHP, her baseline platelet count was normal at 194 thou/cm^2^ (reference: 140-350 thou/cm^2^). Seven days later, she presented to the emergency department (ED) with significant bruising, epistaxis, and diarrhea with melena. Her platelet count at this time was 10 thou/cm^2^, and she received 2 units of platelets in the ED. A week later, her symptomatic thrombocytopenia had resolved with rising platelet counts (Figure 1).

**Table 1:** Neoadjuvant and adjuvant therapy agent, dosage, and date of administration. TCHP: Taxotere® + Carboplatin + Herceptin® + Perjeta®, HER2: human epidermal growth factor receptor 2, AUC: area under the curve, AC: anthracycline and cyclophosphamide.

Neoadjuvant therapy TCHP		
Cycle 1/4		
Day 0	Trastuzumab	8 mg/kg
	Pertuzumab	840 mg
	Docetaxel	75 mg/kg
	Carboplatin	AUC 6
Cycle 2/4		
Day 21	Trastuzumab	6 mg/kg
	Pertuzumab	420 mg
	Docetaxel	15% reduction
	Carboplatin	AUC 5
Cycle 3/4		
Day 48	Pertuzumab	420 mg
	Docetaxel	15% reduction
	Carboplatin	AUC 4
Day 60	Trastuzumab	2 mg/kg
Cycle 4/4		
Day 69	Pertuzumab	420 mg
	Docetaxel	30% reduction
Day 83	Trastuzumab	2 mg/kg
AC neoadjuvant therapy		
Cycle 1/4		
Day 0	Doxorubicin	60 mg/kg
	Cyclophosphamide	600 mg
Cycle 2/4		
Day 14	Doxorubicin	60 mg/kg
	Cyclophosphamide	600 mg
Cycle 3/4		
Day 28	Doxorubicin	60 mg/kg
	Cyclophosphamide	600 mg
Cycle 4/4		
Day 42	Doxorubicin	60 mg/kg
	Cyclophosphamide	600 mg
Anti-HER2 adjuvant therapy		
Day 0	Trastuzumab	2 mg/kg
	Pertuzumab	420 mg IV
Day 36	Trastuzumab	6 mg/kg
	Pertuzumab	420 mg IV
Day 57	Trastuzumab	2 mg/kg
	Pertuzumab	420 mg IV
Day 64	Trastuzumab	2 mg/kg
Day 71	Trastuzumab	2 mg kg
Day 85	Pertuzumab	420 mg IV
Day 92	Trastuzumab	2 mg/kg
Day 99	Trastuzumab	2 mg/kg
Day 106	Pertuzumab	420 mg IV
Day 113	Trastuzumab	2 mg/kg
Day 127	Pertuzumab	420 mg IV
Day 134	Trastuzumab	2 mg/kg
Day 148	Pertuzumab	420 mg IV
Day 168	Pertuzumab	420 mg IV
Day 190	Pertuzumab	420 mg IV
Day 211	Pertuzumab	420 mg IV
Day 232	Pertuzumab	420 mg IV

Prior to beginning her second cycle, her platelet count was normal at 305 thou/cm^2^. Due to the episode of severe thrombocytopenia following her first cycle, her chemotherapeutic regimen was altered: carboplatin was reduced from area under the curve (AUC) of 6 to AUC of 5 and docetaxel was dose reduced by 15%. Full doses of trastuzumab and pertuzumab were given in Table 1. Three days following the second cycle, she presented to the ED with fatigue, body aches, and nonpetechial bruising. She was found to have a significant thrombocytopenia of 5 thou/cm^2^, prompting platelet transfusion with subsequent improvement in her platelet count (Figure 1). Two days later, at her cycle two nadir appointment, mild bruising and a pruritic erythematous papular rash on her face were present. Laboratory studies confirmed a mild thrombocytopenia of 31 thou/cm^2^ (Figure 1). A bone marrow biopsy was performed, showing a normal cellular marrow with active trilineage hematopoiesis without any abnormality. Cytogenetics was normal.

**Figure 2:**
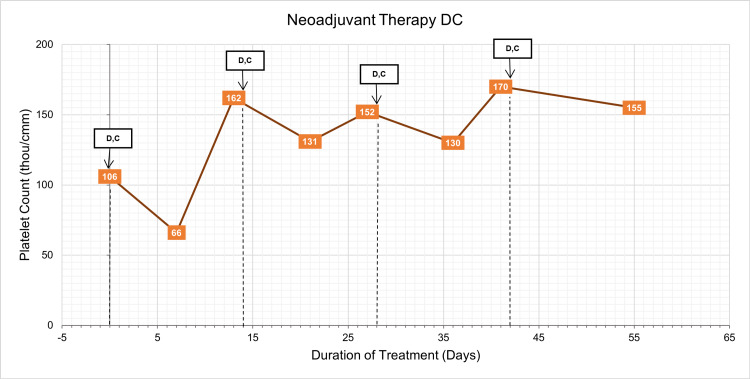
Graph of platelet count over treatment duration with overlayed neoadjuvant therapy timeline with doxorubicin and cyclophosphamide administration. Dashed lines indicate when chemotherapeutic agents were administered and correspond with labels above. D: doxorubicin, C: cyclophosphamide.

Her isolated severe thrombocytopenia without anemia or leukopenia was an unusual adverse effect of TCHP especially as recurrence was undeterred by dose reductions of myelosuppressive chemotherapy. With the possibility that trastuzumab may be causing the thrombocytopenia, trastuzumab administration was omitted for the third cycle and she was treated with 15% dose-reduced docetaxel, AUC 4 carboplatin, and full-dose pertuzumab (Table 1). Her platelet count was followed closely during this cycle. As shown in Figure 2, she experienced mild gradual decreases in her platelet counts over the next week with nadir of 82 thou/cm^2^ on the third day following infusion, but she was asymptomatic and did not require platelet transfusions or evaluation in the emergency department. As planned, she was given trastuzumab two weeks after the Taxotere® + Carboplatin + Perjeta® (TCP) regimen to complete cycle three. Following the infusion, her platelet count fell precipitously from 123 thou/cm2 preceding the infusion to 44 thou/cm2 three days after the infusion. However, she remained asymptomatic during this time (Figure 1).

Given her repeated severe recurrent thrombocytopenia following standard full-dose neoadjuvant chemotherapy, her treatment plan was changed to four cycles of docetaxel and anti-HER2 antibody followed by four cycles of doxorubicin and cyclophosphamide (Table 1). Nine days after trastuzumab infusion, her fourth cycle with full-dose pertuzumab and 30% dose-reduced docetaxel was administered. Her platelet count prior to her fourth cycle was normal at 139 thou/cm^2^ with significant decrease in platelet count of 10,000 thou/cm^2^ seen on lab work and was transfused with 2 units of platelets (Figure 1). Trastuzumab was then given two weeks after with preinfusion platelet count of 175 thou/cm^2^ (Table 1). She remained asymptomatic following infusion with next measured platelet count six days later showing a mild thrombocytopenia of 87 thou/cm^2^ (Figure 1). She then received four cycles of dose-dense doxorubicin and cyclophosphamide. This regimen was well-tolerated without significant thrombocytopenia during this time (Figure 2). After completion of neoadjuvant chemotherapy, she underwent lumpectomy and right axillary sentinel lymph node biopsy. She had complete therapeutic response in both the breast and sentinel lymph nodes without evidence of residual invasive or noninvasive breast cancer.

**Figure 3:**
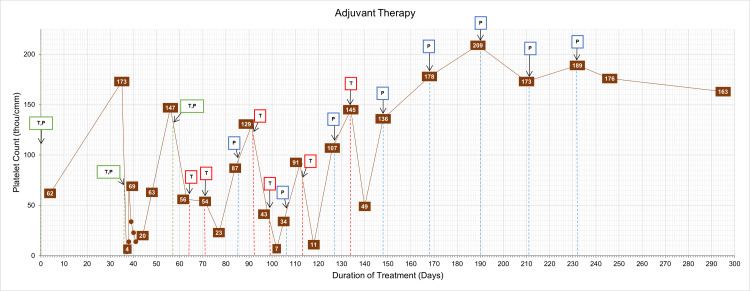
Graph of platelet count over treatment duration with overlayed adjuvant therapy timeline with trastuzumab and pertuzumab administration. Dashed lines indicate when chemotherapeutic agents were administered and correspond with color-coded labels above. Blue is pertuzumab, red is trastuzumab, and green is pertuzumab and trastuzumab. T: trastuzumab, P: pertuzumab.

Standard of care in early-stage HER2/neu-positive breast cancer is a full year of adjuvant trastuzumab and generally pertuzumab, especially if given in the neoadjuvant setting [1]. Even though it seemed likely that her thrombocytopenia was secondary to trastuzumab administration, it was uncertain if a synergistic drug effect was occurring among the cytotoxic chemotherapy given alongside trastuzumab and pertuzumab during the neoadjuvant therapy, amplifying the observed thrombocytopenic events. Given the clinical benefit of adjuvant trastuzumab and pertuzumab [2,3], it was decided to initiate a pharmacological rechallenge with trastuzumab and pertuzumab at the standard three weekly doses with close monitoring of her complete blood count (CBC). Prior to starting adjuvant therapy, her platelet count was normal at 155 thou/cm^2^. However, two days after the first administration of trastuzumab and pertuzumab, she presented to the emergency room with a full-body petechial rash, mucosal bleeding, hematuria, and severe bruising. A CBC revealed a platelet count of 4 thou/cm^2^. Two units of platelets were transfused to which her platelet count appropriately increased (Figure 3).

During the adjuvant therapy course, she received nine total trastuzumab infusions as detailed in Table 1 with each infusion causing dramatic decreases in her platelet counts as detailed in Figure 3. During this treatment period with trastuzumab and pertuzumab, she continued to have episodes of symptomatic thrombocytopenia with petechiae and occasional epistaxis. She was closely monitored in the outpatient setting following infusions. Multiple dosing adjustments were made, including separating trastuzumab and pertuzumab administration temporally and administering them independently. Given the repeated severe intolerance of trastuzumab, trastuzumab was discontinued and treatment with independent full-dose pertuzumab was continued every three weeks and she completed her full course of adjuvant therapy (Table 1). Her platelet counts stabilized, and she did not experience any further episodes of severe thrombocytopenia during adjuvant monotherapy with pertuzumab (Figure 3). She completed adjuvant radiation therapy and was started on adjuvant treatment with tamoxifen. Today, she is doing well without recurrence of cancer for four years since finishing treatment.

## Discussion

To our knowledge, this is the eleventh reported case of trastuzumab-induced thrombocytopenia to date [4-13].

Temporal relationship between trastuzumab and thrombocytopenia development

Given the severity and timing of her recurrent thrombocytopenia, docetaxel and carboplatin were unlikely culprits. While docetaxel and carboplatin do cause thrombocytopenia [14,15], they typically occur between 7 and 10 days after administration, only cause modest reductions in platelet count, and are responsive to dose reduction. Trastuzumab-induced thrombocytopenia occurs within hours to days [4-13], with a maximum reported time to onset eight days [11]. In one case, trastuzumab-induced thrombocytopenia did not occur until the eighth cycle [12]. While cases where patients were rechallenged with trastuzumab generally showed recurrence of thrombocytopenia [7-10,12], one reported case showed normal platelet counts after the fourth infusion of trastuzumab [9].

In our patient’s case, there was a temporal relationship and almost cause-and-effect association between the initiation of trastuzumab and development of severe thrombocytopenia. This was a diagnostic challenge during her treatment because of the concurrent infusion of neoadjuvant cytotoxic chemotherapy. Using validated scoring systems to determine the likelihood of trastuzumab causing this adverse reaction, this event scored as “probable” (6/12) on the Naranjo scale [16]. We put forth a case where we can clearly delineate trastuzumab-induced thrombocytopenia through neoadjuvant and adjuvant chemotherapy. Importantly, all laboratory evaluations described in this report were drawn and processed by the same facility each time at our tertiary medical center. Standard protocols were followed to ensure that the recorded thrombocytopenia was not secondary to collection error or pseudothrombocytopenia.

Management of trastuzumab-induced thrombocytopenia

Reported cases have described success in treating trastuzumab-induced thrombocytopenia similarly to immune thrombocytopenic purpura (ITP). Patients demonstrated successful recovery of platelet counts with immunosuppressive treatment with either intravenous immunoglobulins (IVIG), high-dose steroids, or both together [4-10,12,13]. Other reports have faced similar diagnostic challenges due to concurrent cytotoxic chemotherapy infusion and have successfully demonstrated platelet count recovery with platelet transfusion [6,7,10,12,13], thrombopoietin (TPO) [8,13], granulocyte-macrophage colony-stimulating factor (GMCSF) [11], and recombinant interleukin (IL) [11,13], or etamsylate [11]. Because of our patient’s concurrent cytotoxic chemotherapy infusion, initial drops in platelet count were treated as chemotherapy-induced bone marrow suppression. Therefore, our patient was treated with therapeutic platelet infusions instead of immunosuppression. In all cases except for a reported case where the trastuzumab-induced thrombocytopenia resolved [9], trastuzumab was either discontinued or patients experienced progression of disease [4-8,10-13]. Our patient did not experience cessation of thrombocytopenia with repeated trastuzumab infusion; however, the perceived benefit of anti-HER2 therapy in preventing disease relapse outweighed the risk of withholding treatment [1-3]. For this reason, she was maintained on pertuzumab monotherapy for the remaining duration of the one year of adjuvant therapy and trastuzumab was permanently discontinued.

Pertuzumab as monotherapy

Pertuzumab monotherapy has been poorly described in the literature to date. The original phase I trial of pertuzumab in 21 patients with an assortment of advanced cancers only demonstrated partial responses in two patients: one patient with ovarian cancer and the other with islet cell carcinoma of the pancreas [17]. Of note, the three patients with breast cancer in this cohort were HER2 negative and showed no clinical response [17]. In patients with metastatic, HER2-negative breast cancer, only six of 78 patients responded or had stable disease for six months or more and median time to progression was reported to be between 43 and 44 days [18]. In patients with HER2-positive breast cancer that progressed during trastuzumab therapy, pertuzumab monotherapy demonstrated an objective response and clinical benefit in a small percentage of patients that was significantly lower than those who received dual pertuzumab and trastuzumab therapy [19].

Standard adjuvant therapy for HER2-positive breast cancer is one year of trastuzumab and pertuzumab together [1]. Though the literature available suggests that pertuzumab monotherapy is inferior to dual anti-HER2 therapy in advanced breast cancer, there are not any data available to suggest the superiority of dual therapy to pertuzumab monotherapy in the adjuvant setting of HER2-positive breast cancer. In the setting of nonhematologic toxicity, pertuzumab and trastuzumab are generally held together. Cathomas and von Moos describe a case of a patient with trastuzumab-induced thrombocytopenia who was successfully managed with lapatinib anti-HER2 therapy instead of trastuzumab [5]. The patient described in this report is still in remission, demonstrating that adjuvant pertuzumab monotherapy is a viable option for patients with HER2-positive breast cancer who are intolerant of trastuzumab.

## Conclusions

Trastuzumab is a mainstay of treatment in HER2-positive breast cancer but may cause severe thrombocytopenia in a small subset of patients. We present a case report of HER2-positive breast cancer that was complicated by severe thrombocytopenia following trastuzumab administration with definitive improvement following discontinuation of trastuzumab. Our study contributes to the body of work describing a rare, but severe, side effect of a commonly used chemotherapeutic agent while providing evidence of successful solitary use of pertuzumab as maintenance therapy. Given that pertuzumab is typically only used in addition to trastuzumab, evidence of its successful independent use is of clinical value to patients with trastuzumab intolerance.
